# In Vitro Characterization of Neutralizing Hen Antibodies to *Coxsackievirus* A16

**DOI:** 10.3390/ijms22084146

**Published:** 2021-04-16

**Authors:** Pharaoh Fellow Mwale, Chi-Hsin Lee, Peng-Nien Huang, Sung-Nien Tseng, Shin-Ru Shih, Hsin-Yuan Huang, Sy-Jye Leu, Yun-Ju Huang, Liao-Chun Chiang, Yan-Chiao Mao, Wei-Chu Wang, Yi-Yuan Yang

**Affiliations:** 1 Ph.D. Program in Medical Biotechnology, College of Medical Science and Technology, Taipei Medical University, Taipei 110301, Taiwan; pmwale@medcol.mw (P.F.M.); chihsine@msn.com (C.-H.L.); 2 School of Medical Laboratory Science and Biotechnology, College of Medical Science and Technology, Taipei Medical University, Taipei 110301, Taiwan; peter2003316@yahoo.com.tw (H.-Y.H.); jonsauwi@tmu.edu.tw (Y.-J.H.); 3 Division of Infectious Diseases, Department of Pediatrics, Linkou Chang Gung Memorial Hospital, Research Center for Emerging Viral Infections, Chang Gung University, Taoyuan 333423, Taiwan; mark1005@gmail.com; 4 Research Center for Emerging Viral Infections, Chang Gung University, Taoyuan 333323, Taiwan; hermit01tw@yahoo.com.tw; 5 Department of Laboratory Medicine, Linkou Chang Gung Memorial Hospital, Research Center for Emerging Viral Infections, Chang Gung University, Taoyuan 333423, Taiwan; srshih@mail.cgu.edu.tw; 6 Department of Microbiology and Immunology, School of Medicine, College of Medicine, Taipei Medical University, Taipei 110301, Taiwan; cmbsycl@tmu.edu.tw; 7 Graduate Institute of Medical Sciences, College of Medicine, Taipei Medical University, Taipei 110301, Taiwan; xfreestylew@gmail.com; 8 Institute of Bioinformatics and Structural Biology, College of Life Sciences, National Tsing Hua University, Hsinchu 300040, Taiwan; axe956956@gmail.com; 9 Division of Clinical Toxicology, Department of Emergency Medicine, Taichung Veterans General Hospital, Taichung 407219, Taiwan; doc1385e@gmail.com; 10 Core Laboratory of Antibody Generation and Research, Taipei Medical University, Taipei 110301, Taiwan

**Keywords:** *Coxsackievirus* A16 (CA16), single-chain variable fragment (scFv), phage display libraries, *Enterovirus* 71 (EV71), immunoglobulin yolk (IgY)

## Abstract

*Coxsackievirus* A16 (CA16) is one of the major causative agents of hand, foot, and mouth disease (HFMD). Children aged <5 years are the most affected by CA16 HFMD globally. Although clinical symptoms of CA16 infections are usually mild, severe complications, such as aseptic meningitis or even death, have been recorded. Currently, no vaccine or antiviral therapy for CA16 infection exists. Single-chain variable fragment (scFv) antibodies significantly inhibit viral infection and could be a potential treatment for controlling the infection. In this study, scFv phage display libraries were constructed from splenocytes of a laying hen immunized with CA16-infected lysate. The pComb3X vector containing the scFv genes was introduced into ER2738 *Escherichia coli* and rescued by helper phages to express scFv molecules. After screening with five cycles of bio-panning, an effective scFv antibody showing favorable binding activity to proteins in CA16-infected lysate on ELISA plates was selected. Importantly, the selected scFv clone showed a neutralizing capability against the CA16 virus and cross-reacted with viral proteins in EV71-infected lysate. Intriguingly, polyclonal IgY antibody not only showed binding specificity against proteins in CA16-infected lysate but also showed significant neutralization activities. Nevertheless, IgY-binding protein did not cross-react with proteins in EV71-infected lysate. These results suggest that the IgY- and scFv-binding protein antibodies provide protection against CA16 viral infection in in vitro assays and may be potential candidates for treating CA16 infection in vulnerable young children.

## 1. Introduction

*Coxsackievirus* A16 (CA16) is one of the predominant etiological causative agents of several epidemics associated with hand, foot, and mouth disease (HFMD) in young children and infants [1,2]. The disease is generally mild; however, it may lead to neurological complications, such as aseptic meningitis, and in serious cases death may occur [3,4]. Outbreaks of HFMD have been recorded in the past in major continents such as Europe, mainly England and Wales [5], and in Asian countries, such as Taiwan from 1998 to 2006 [6]. Despite CA16′s association with many HFMD cases, co-infections with other enterovirus strains, including enterovirus 71 and coxsackieviruses A6 and A10, have occurred, resulting in viral recombination, which increases the difficulty of controlling HFMD epidemics [7]. However, a report from China indicates that some outbreaks of HFMD may be caused by strains with genetic recombination [8]. Such viral strains are difficult to treat, further raising public health concerns. No effective vaccines or antiviral drugs that target CA16 infections are available. Currently, two vaccines (formalin-inactivated EV71 vaccines) have been approved in China by the National Medical Products Administration (NMPA), while some vaccine candidates are still in clinical trials [9,10]. However, the recently approved vaccines cannot cross-react against HFMD-associated *coxsackievirus*, such as CA16, because the vaccine was developed to target EV71 strains [11]. Surprisingly, the vaccine was not used worldwide. To combat the epidemic of HFMD, more antiviral drugs or antibodies will be needed. Therefore, production of efficacious neutralizing antibodies against CA16 infection may be of great importance. Production of new vaccines targeting new and emerging strains is a challenging endeavor that requires extensive time. Strains can quickly mutate, reducing the effectiveness of previous treatments. Moreover, antibody-based products have shown potential against new infectious diseases [12]. Hence, developing antibodies could impact the new crop of infectious diseases.

CA16 is very contagious and is transmitted via respiratory secretions or feces infection. Typically, the virus remains viable in the respiratory tract for up to three weeks or in feces for up to eight weeks after initial infection [13]. As different viruses can contribute to HFMD, reoccurrence of the disease is possible. Further, person-to-person transmission can occur due to unwashed hands or surfaces soiled by feces. CA16 and other strains (EV71) are small viruses with positive single-stranded sense genomic ribonucleic acids (RNAs). The CA16 and EV71 RNAs are protected by a non-enveloped icosahedral capsid built from pentamers, each of which consists of four structural proteins, namely VP1, VP2, VP3, and VP4. In general, these structural viral proteins are found in *Enteroviruses* and other related *Coxsackieviruses*, where VP1, VP2, and VP3 form major structural proteins and important antigens [14]. VP4, on the other hand, forms the inner lining of the viral capsid [15]. Therefore, VP1 is believed to contain the site for receptor attachment [16]. Thus, VP1 is a viable target for preventing CA16 or other enteroviral infections.

Single-chain fragment variable (scFv) was first described and constructed more than three decades ago by Bird et al. [17] and Huston et al. [18]. Since then, selections of genetically engineered scFvs have been seen as effective therapeutics in many diseases such as cancer, influenza, and enteric colibacillosis [19,20,21,22]. ScFv antibody consists of a variable region of heavy chain (V_H_) and light chain (V_L_) joined by an adjustable peptide linker. It is a small recombinant antibody with a molecular weight ranging from 26 to 30 kilodaltons (kDa). In contrast to full IgG molecules, the scFv antibody maintains its antigen-binding ability, despite lacking the constant regions, and could be genetically manipulated to enhance binding activities and specificity [23]. Therefore, scFv antibodies have demonstrated neutralization of various viruses, blocking viral infection and even protecting hosts from infectious disease [24,25]. These described studies imply that scFv antibodies may act as a potential therapeutic treatment and diagnostic agent for virus infections such as CA16.

In this study, we generated and characterized the immunoglobulin yolk (IgY)- and scFv-binding protein antibodies against proteins from CA16-infected lysate by using two rounds of PCR to construct a full-length scFv gene and then express its protein, determine its binding affinity, and demonstrate cross-reactivity and an antiviral neutralization effect in vitro. The scFv-binding protein was selected by phage display technology after passive immunization in a laying hen.

## 2. Results

### 2.1. Characterization of Polyclonal Immunoglobulin Yolk (IgY)-Binding Protein Antibodies

**Figure 1:**
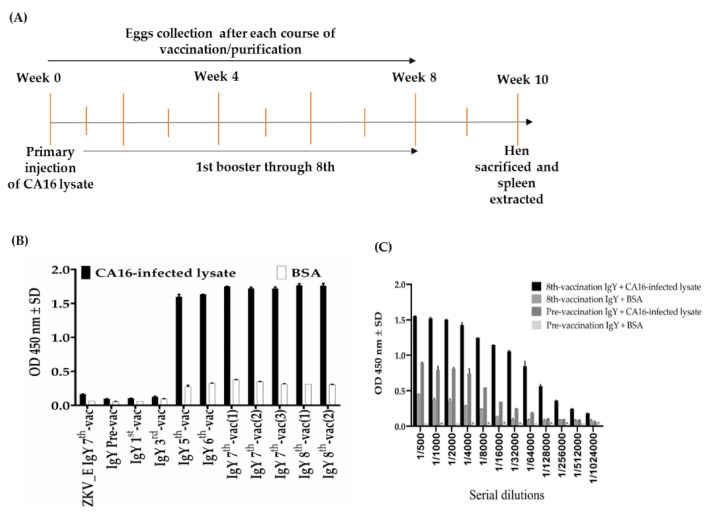
Immune response analysis before each immunization. (**A**) Simplified diagram of vaccination protocol with primary injection, then first booster through booster number 8. (**B**) The binding ability of purified immunoglobulin yolk (IgY)-binding protein antibodies against CA16-infected lysate and bovine serum albumin (BSA). The abbreviations pre- or 8th-vac, etc., represent pre-vaccination through to the eighth vaccination on a weekly basis. The numbers 1, 2, or 3 mean the number of eggs IgY purified at that vaccination. The ELISA assay was run to confirm the immune response induction in the laying hen. Zika virus envelope (ZKV_E) IgY 7th-vac was used as a negative antibody control. (**C**) An ELISA test was conducted to reveal the total IgY antibody at dilutions of 1/500 to 1/1,024,000. The assay compared the pre-vaccination IgY titer to the 8th-vaccination IgY titer. The experiments were run in duplicate and data presented as average ± standard deviation (SD).

**Figure 2:**
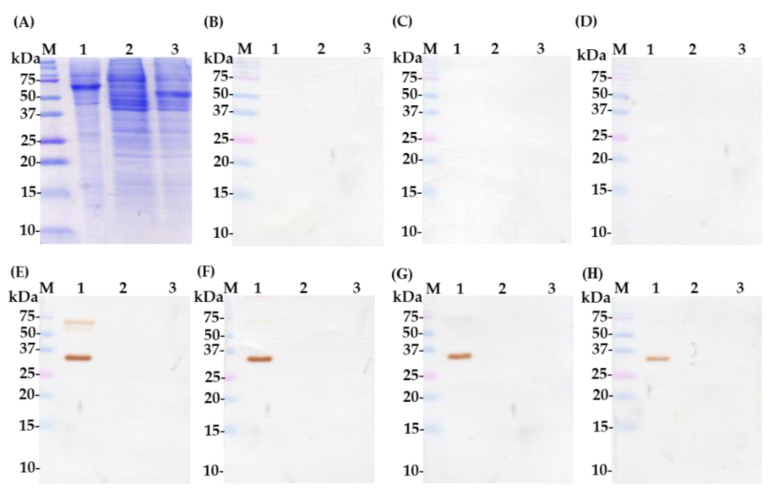
Characterization of immunoglobulin yolk-binding protein by Western blot. SDS-PAGE displaying CA16-infected lysate (lane 1), non-infected (RD cells) lysate (lane 2), and BSA (lane 3) stained with Coomassie blue dye; lane M: protein marker (**A**). The samples were transferred onto PVDF membranes and detected with pre-vaccination IgY (**B**); 1st-vaccination IgY (**C**); 3rd-vaccination IgY (**D**); 5th-vaccination IgY (**E**); 6th-vaccination IgY (**F**); 7th-vaccination IgY (**G**); and 8th-vaccination IgY (**H**) antibodies at a dilution of 1:3000. The complex was then revealed by HRP-conjugated donkey anti-chicken IgY antibodies at a dilution of 1:10,000. BSA and non-infected lysate were used as negative antigen controls.

A white laying hen was vaccinated intramuscularly on the thigh at a different site with CA16-infected lysate. The hen was vaccinated for 8 weeks as shown in Table 1. The eggs were collected from the hen before and after vaccination every 7 days for IgY extraction and purification and then, after another 7 days from 9 weeks, the hen was sacrificed (Figure 1A). The IgY purification was done according to the published protocol with few changes [26]. To evaluate induced immune response in the hen, we conducted an ELISA assay to test an increased antibody response during the 8-week vaccination period. Figure 1B shows the relatively high immune response at week 3 after a booster dose of CA16-infected lysate. The titers slowly increased up to week 6, then became a plateau thereafter. In addition, the IgY titer was performed by ELISA assay. The eight-vaccination IgY titer was deemed as the highest dilution at 1/32,000 dilutions, where optical density value was equal to or greater than 1.0 compared to the pre-vaccination IgY, which showed a low titer (optical density less than 1.0) at a dilution of 1/200 (Figure 1C). Further, we performed SDS-PAGE (Figure 2A) and Western blot assay (Figure 2B–H) to determine the recognition of purified IgY-binding protein antibodies by proteins in CA16-infected lysate. The results showed no protein band signals with pre-, first-, and third-vaccination IgY antibodies (Figure 2B–D) and protein band signals of approximately 35 and 65 kDa with fifth-, sixth-, seventh-, and eight-vaccination IgY (Figure 2E–H). Therefore, the analysis suggests that a strong humoral immune response was achieved in the inoculated hen.

### 2.2. Cloning of Single-Chain Variable Fragment (scFv) Gene

**Figure 3:**
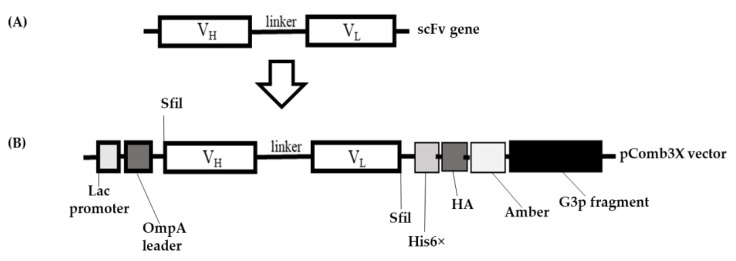
Schematic overview of single-chain variable fragment (scFv) gene cloning into the pComb3X plasmid. The arrangement of the V_H_ and V_L_ regions joined by a flexible linker representing the anti-CA16 scFv gene (**A**). The pComb3X gene constructs contained histidine (His6×) tag and hemagglutinin (HA) tag for detection and purification of recombinant scFv antibodies (**B**). The g3p fragment was included for the phage to display the scFv antibody protein on the surface of the viral particles.

The designed variable regions of the heavy chain (V_H_) and light chain (V_L_), joined by a peptide linker to form a full-length scFv gene (Figure 3A), were ligated to the pComb3X vector (Figure 3B). The V_H_ and V_L_ were PCR-amplified using specific hen primers (primers listed in the method section) [27]. The recombinant DNAs were transformed and then amplified in *Escherichia coli* for use in phage library construction.

### 2.3. Characterization of Phage-Displaying scFv Libraries and Selection of scFv-Binding Protein Clones

**Figure 4:**
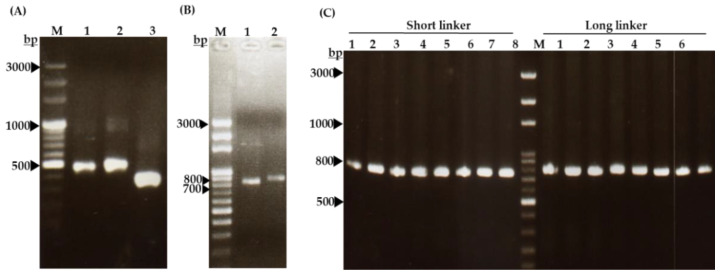
Construction analysis of the scFv-binding protein antibodies phage display libraries and insert confirmation. PCR products of lane 1: V_H_-S (variable region of heavy chain with short-linker); lane 2: V_H_-L (variable region of heavy chain with long-linker); and lane 3: V_L_ (variable region of light chain) (**A**). PCR products of the scFv-S (lane 1) and scFv-L (lane 2) (**B**). PCR amplification of full-length scFv genes from the phage display library for confirming insertion frequency (**C**). Lanes 1–8: scFv genes containing V_H_-peptide linker-V_L_ fragments with short- or long-linkers, respectively. All three panels have DNA ladder (lane M) as a reference point.

**Figure 5:**
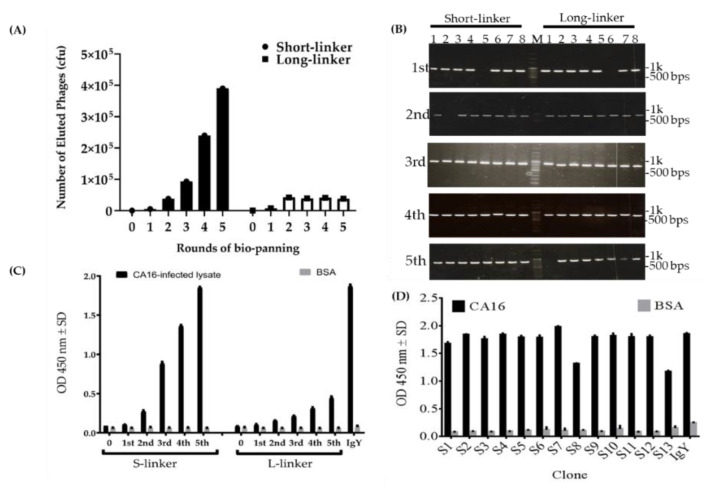
The phage titers, colony-PCR, scFv selection, and their specific binding abilities. (**A**) The recombinant eluted phage titer was checked after the first cycle of panning and throughout the five cycles. Their eluted titer was demonstrated using a colony formation assay. (**B**) After each cycle of the bio-panning process, colonies were randomly selected to confirm the phagemid DNA of the binders; DNA ladder (M) was used for a reference point. (**C**) Amplified phage-binders from each cycle of bio-panning were tested for their binding activities. These eluted titers and their abilities to bind were compared with phages from original libraries (0). (**D**) The ELISA result showed the binding ability of individual clones with short-linkers. IgY-binding protein antibody (1:3000 dilution) was included as a positive control. HRP-conjugated rabbit anti-mouse IgG and HRP-conjugated donkey anti-chicken IgY antibodies were used to confirm the complex at 1:10,000 dilutions, respectively.

To obtain scFv-binding protein antibody libraries, the laying hen was sacrificed 14 days after the last vaccination and then total RNA was isolated from the splenocytes for library construction of the scFv-binding protein antibodies. The results showed that V_H_ gene products were amplified as 400 base pairs (bps) containing short-linkers (Figure 4A, lane 2) or 450 bps containing long-linkers (Figure 4A, lane 3), in the first PCR. On the other hand, the V_L_ genes were also PCR-amplified with the size of approximately 350 bps (Figure 4A, lane 4). Furthermore, the results displayed the amplified V_H_ and V_L_ products that were linked and extended by overlapping PCR to make full-length scFv gene fragments with approximately 750 bps (Figure 4B). The scFv gene in the pComb3X vector was verified by performing colony PCR, which was run using published specific primers [28]. Figure 4C shows 16 clones picked randomly from the plate of each library, which contains the right size of full-length scFv genes to validate the efficiency of the constructed-library (Figure 4C). To further select scFv-binding protein clones with high binding affinity from the two constructed antibody libraries, five cycles of bio-panning steps were conducted; the results are displayed in Figure 5.

The data showed a sharp increase of eluted phage titer in the third to fourth rounds, then another skyrocketing increase to the fifth round in the short-linkers; meanwhile, in the long-linkers no increase of titer was observed (Figure 5A). This implies that the eluted phage-binders with the short-linkers were successful compared to binders with the long-linkers. Further analysis of bio-panning work was assessed. Colony PCR was run to confirm the positive phage-binders. Figure 5B showed at least 90% positive colonies in both linkers, despite low eluted phage titer in the long-linkers (Figure 5B). We do not know exactly why we experienced such a discrepancy in titer. However, the results validate that the binders against proteins in CA16-infected lysate were eluted. Next, we performed the phage ELISA to evaluate the results of panning through the analysis of the amplified phage titer. The result displayed a similar raise pattern in the short-linkers as those observed with eluted phage titer. The same results were also noticed with the long-linkers as were shown with the eluted phage titer in the long-linkers; however, we observed a slight increase in the fifth round of panning in the long-linkers (Figure 5C). Therefore, this data demonstrates the achievement of amplifying the phage-binders with short-linkers. IgY-binding protein was used as a positive control, while the library phage was used as a negative control (Figure 5A,C). Another ELISA assay was run to determine the binding activities of randomly selected scFv clones (Figure 5D). After the fifth round of bio-panning, a total of 26 colonies were randomly selected from the plate of Luria Bertani (LB) agar. To test their binding activities, the scFv antibody proteins were expressed in Escherichia coli bacteria. The analysis revealed that all 13 scFv clones with short-linkers had strong binding activities. However, no scFv antibodies with long-linkers were identified using the same bio-panning procedure (data not shown). BSA was used as a negative antigen control whereas IgY-binding protein was used as a positive antibody control. Taken altogether, the data suggested that the phage-displaying scFv was enriched in the fifth cycle of bio-panning with short-linkers but not with long-linkers, and also that the scFv clones selected with the short-linkers had strong binding abilities.

### 2.4. Characterization of scFv Antibody after Fifth Cycle of Bio-Panning

**Figure 6:**
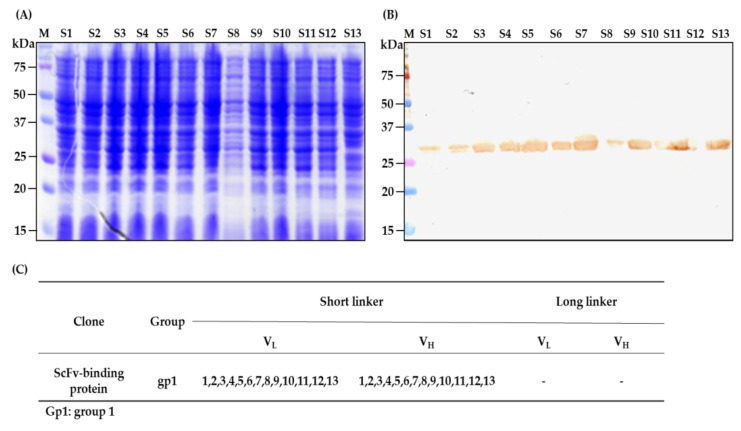
Expression and grouping analysis of scFv antibody. (**A**) Thirteen randomly selected scFv clones were loaded onto respective wells of SDS-PAGE. (**B**) The PVDF membrane was probed by mouse anti-HA tag antibodies at 1:5000 dilutions, then detected by HRP-conjugated rabbit anti-mouse IgG antibodies at 1:5000 dilutions. The expected scFv molecular mass was about 26 kDa. (**C**) All the scFv antibodies had the same amino acid sequences and were classified as one group.

To obtain recombinant antibodies, the total DNA of the phages after the fifth cycle of scFv selection was extracted from bacterial pellets, purified, and transformed into TOP10F’ *Escherichia coli* (competent cells prepared in our laboratory). The SDS-PAGE (Figure 6A) and Western blot (Figure 6B) were performed to confirm the scFv protein expression in *Escherichia coli*. The results demonstrated that all 13 clones with short-linkers were expressed (Figure 6B), while the clones with long-linkers were not assessed because they exhibited a low titer during the colony polymerase chain reaction assay and ELISA phage analysis (data not shown). Interestingly, all the clones had the same amino acids when compared to the hen immunoglobulin germline; therefore, based on their amino acid similarities and differences, the scFv clones with short-linkers were categorized as one group and designated as scFv-binding protein S1 (Figure 6C). When the amino acid of the scFv-binding protein S1 antibody and the germline gene were compared, the results displayed high mutation rates, especially in complementarity determining regions (CDRs), with 41.94% in the light chain and 59.46% in the heavy chain in contrast to framework regions (FRs), which had 10.25% in the light chain or the 6.90% in heavy chain (Figure 6D and Table 2). 

### 2.5. Neutralization Effect of IgY- and scFv-Binding Protein Antibodies

**Figure 7:**
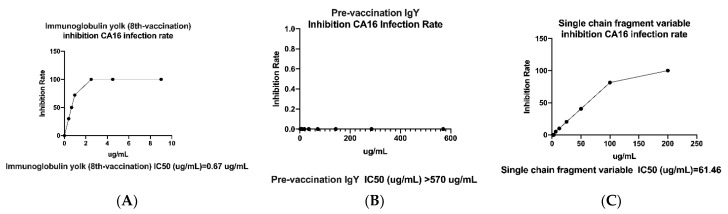
Analysis of IgY and scFv neutralizing activity against coxsackievirus A16. (**A**) Pre-vaccination IgY as negative control antibody with an IC_50_ greater than 570 µg/mL. (**B**) Immunoglobulin yolk (8th vaccination) antibody with an IC_50_ equal to 0.67 ± 0.06 µg/mL. (**C**) Scheme 50. equal to 61.46 ± 1.75 µg/mL. All experiments were carried out in triplicate. The results are presented as means plus or minus standard deviation.

Polyclonal immunoglobulin yolk antibody and single-chain fragment variable antibody (S1 clone) were tested for whether they could inhibit CA16 viral infection; an ELISA plaque in vitro was conducted. The assay was run in triplicate, and included antibody negative control as pre-vaccination IgY with the calculated half maximal inhibitory concentration (IC_50_) of greater than 570 µg/mL (Figure 7A). Also, the IgY antibody was able to neutralize viral infection of RD cells with the IC_50_ of 0.67 ± 0.06 µg per mL (Figure 7B), whereas scFv showed a minimum of 61.46 ± 1.75 µg per mL (Figure 7C). However, no neutralization activity was observed for the negative control. The data suggest that IgY has strong neutralization potential for CA16 viral infection in vitro compared to scFv-binding protein S1 and control antibodies.

### 2.6. Determination of Cross-Reactivity of scFv-Binding Protein S1 and IgY against CA16- and EV71-Infected Lysate and BSA

**Figure 8:**
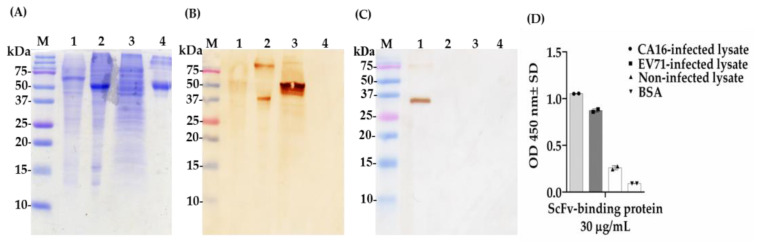
Cross-reactive analysis of scFv and IgY antibodies against coxsackievirus A16 and enterovirus 71. (**A**) SDS-PAGE showing protein marker (lane M); CA16-infected lysate (lane 1); EV71-infected lysate (lane 2); non-infected lysate (lane 3); BSA protein (lane 4). Western blot assay probed with (**B**) scFv-binding protein S1 and (**C**) IgY antibodies. (**D**) The ELISA test was carried out by the addition of scFv antibody and mouse anti-HA tag, followed by HRP-conjugated rabbit anti-mouse IgG antibodies. The BSA was included as an antigen control on both the Western blot and ELISA assays.

Next, we further conducted Western blot and ELISA assays to investigate the cross-reaction between coxsackievirus and enterovirus. Surprisingly, after the transfer of viral proteins from SDS-PAGE (Figure 8A) to the membrane and a probe with antibodies, we found that scFv-binding protein S1 had the ability to recognize two different protein bands in EV71-infected lysate around 37 or 75 kDa; a smear in CA16-infected lysate; and a strong band in non-infected lysate (RD cell lysate) around 50 kDa, though no band was observed in the BSA sample in Western blot assays (Figure 8B). Intriguingly, the IgY antibody purified after the eight vaccination recognized two protein bands of different sizes, approximately 35 and 75 kDa, respectively (Figure 8C). The result of the ELISA assay showed that scFv-binding protein S1 had a strong binding ability against proteins in CA16-infected lysate with an optical density (OD) greater than 1.0 and moderate binding activity against proteins in EV71-infected lysate; a weak binding affinity was observed against proteins in non-infected lysate and the BSA sample with OD less than 0.3 (Figure 8D). This data suggest that scFv-binding protein S1 cannot recognize specific proteins of coxsackievirus A16; rather, it has cross-reactive abilities against proteins in EV71-infected lysate and cellular proteins, whereas polyclonal IgY antibody can specifically recognize proteins in CA16-infected lysate with no cross-reactive ability, which is good for CA16 diagnosis.

## 3. Discussion

The goal of this study was to generate and characterize neutralizing single-chain fragment variable and immunoglobulin yolk antibodies against proteins in CA16-infected lysate. CA16 and other enteroviruses have been associated with several outbreaks of hand, foot, and mouth disease across the major continents [29]. In this study, we used phage display technology for scFv antibody selection against CA16-infected lysate that was made from infected human rhabdomyosarcoma (RD) cells. This technique is now used by most research scientists for antibody screening against target antigens, which requires the construction of an antibody library from an immunized animal’s splenocytes, against which the specific antibodies are screened [30]. For this purpose, we first vaccinated a laying hen with CA16-infected lysate (Table 1 and Figure 1A), then monitored the immune response weekly and analyzed the IgY antibodies titer using an ELISA assay. The results showed a sharp increase from week 5 followed by plateau formation at weeks 7 and 8. The hen did not lay eggs in weeks 2 and 4; the exact reasons why this happened are unknown, but it is speculated that it could be the stress of intramuscular injection. Also, the immune response could be elicited in week 4 (Figure 1A,B). However, we observed a lower optical density, below 1.0, at the dilution of 1/64,000 against the target antigen, but the titer was still significantly high despite being serially over-diluted (1/64,000) (Figure 1C). On the other hand, we also observed positive results in the Western blot assay (Figure 2A–H), suggesting that the immune response was strongly induced in the immunized hen. A previous study with the same type of vaccination strategy in an egg-laying hen showed a different pattern of titer [31]; this is because the use of different viral strains may produce different immunogenicities. In their study, the authors observed high levels and a specific anti-influenza virus IgY antibodies titer after the fifth vaccination, reaching a climax after the seventh vaccination. This result supports the induction of the immune response against the viral proteins in hen laying and is feasible despite the use of different strains as an antigen. Next, the cloning of the full-length scFv gene in the pComb3X vector using published specific primers was achieved (Figure 3A,B). Following cloning results, two antibody libraries were constructed that contained 4 × 10^6^ and 5 × 10^6^ plaque-forming units (pfu). The phage library constructed after the variable region of the immunoglobulin gene was amplified to make a full-length scFv gene, then later was ligated to the phagemid vector and analyzed by agarose gel electrophoresis to confirm the amplified PCR products (Figure 4A–C). These data suggests that the phage-displaying scFv antibody was successfully achieved. Moreover, the technique is a high throughput for the selection of diagnostic and therapeutic scFv antibody candidates. Therefore, we further conducted colony PCR to check the phage containing the scFv genes, then phage ELISA after bio-panning to demonstrate the binding activities of the scFv-binders, and we also performed a test of the individual binding ability of the selected scFv clones. The data showed a gradual increase in phage-displaying scFv binders with short-linkers in the fifth round of the bio-panning process. There were no binding activities for the scFv-binders with long-linkers, and the clones were also confirmed to have scFv genes, all of them with both short- and long-linkers (Figure 5A–C). These results suggest that scFv antibodies were effectively enriched at this point in the last cycle of antibody selection. A similar pattern of results was obtained in previously published data, although the scFv antibodies were enriched in the fourth cycle of the bio-panning process [32], of which six groups neutralizing anti-venom scFv antibodies were screened. This further proves that the technique is novel and powerful in the generation of target-specific scFv-binding protein antibodies. All 13 randomly selected scFv clones were successfully expressed in the TOP10F’ *Escherichia coli* system (Figure 6A,B). This implies that the selection method was effective. Furthermore, after five cycles of bio-panning, the scFv clones were sequenced; one group of the clones was identified (Figure 6C) and, from it, a high-affinity scFv (scFv-binding protein S1) was chosen for further characterization. The predicted amino acid sequences of the scFv-binding protein S1 clone were aligned and analyzed by comparison to the laying-hen immunoglobulin germline. The analysis indicated that the variation in the complementary determining regions was significantly greater than that in the framework regions (Table 2), representing 10.25% in FRs and 41.94% in CDRs of the light chain and 6.90% in FRs and 59.46% in CDRs of the heavy chain. Overall, the data strongly showed a high mutation rate in CDRs, the region responsible for binding as far as the antibody is concerned. This, however, revealed that the lengths of CDR3 in the V_L_ and V_H_ were influenced by the presence of amino acid insertions in the regions. As it is generally believed that CDRs contribute to the binding specificity to a specific epitope, differences in the diversity of CDRs in the V_L_ and V_H_ regions may also reflect the different binding epitopes of this scFv. This is why the different binding abilities pattern was observed between the monoclonal scFv clone and polyclonal IgY antibodies. The specific binding activity of scFv-binding protein S1 was further assessed. The pre-vaccination IgY did not neutralize the CA16 virus (Figure 7A), which is not surprising because its titer was low compared to the last vaccination IgY (eight vaccination). The results, on the other hand, showed that IgY- and scFv-binding protein antibodies inhibit CA16 virus infection in RD cells. According to the results, the IgY antibody has high potency toward the CA16 virus as compared to the scFv antibody, which needed at least 0.65 µg / mL to neutralize the virus (Figure 7B), whereas the IgY antibody required at least 60 µg per mL to combat the CA16 virus (Figure 7C). On the other hand, the IgY antibody demonstrated no cross-reactivity with proteins in EV71-infected lysate, but showcased a specific binding ability against proteins in CA16-infected lysate (Figure 8A–D). It detected the proteins of which the molecular weights were approximately 35 kDa and also 75 kDa, which represent VP1 and 3CD (polyprotein) according to published data [33]. A similar detection pattern was observed during a probe with scFv antibody against EV71-infected lysate. However, we do not know why the scFv-binding protein antibody recognized cellular proteins in a mock sample. Previous studies have also demonstrated the possibility of the above-described results, when scFv and polyclonal antibodies were used, respectively, against different enterovirus antigens [34,35]. The results showed significant binding of the scFvs to viral proteins; however, they partially neutralized the viral infections of CA16 and influenza type A viruses. This is contrary to our data, which revealed the significant inhibition of viral infection to RD cells. For instance, most single-chain antibodies have the potential for both detection and neutralization effects [36,37]. Despite the scFv antibody not being able to detect the viral proteins in CA16-infected lysate, it did prove more potent at inducing protection of RD cells against CA16 virus infection in vitro. These data suggest that neutralizing scFv-binding protein antibody could be a promising and effective treatment for CA16 infections and could also be used to prevent CA16 HFMD outbreaks. However, the activity of scFv-binding protein antibody may not entirely prove the direct binding with CA16 virus, due to the possibility of the antibody binding to cellular proteins that are vital for the virus’s life cycle. Therefore, it is essential that our hen-derived scFv-binding protein antibody be further validated for its neutralization effects in an in vivo model.

## 4. Materials and Methods

### 4.1. Ethics Declaration

The hen experiment in this work was performed following the guidelines of the Taipei Medical University Animal Center. The animal use protocol was reviewed and approved by the animal care and use committee of the Institution (IACUC approval number LAC-2015-0302, 28 March 2016).

### 4.2. Cells, Viruses, and Bacteria

The human rhabdomyosarcoma (RD) cells were grown in Dulbecco’s modified eagle medium (DMEM) supplemented with 10% of fetal bovine serum (FBS) (Fisher Scientific, Fair Lawn, NJ, USA) and with 5% CO_2_ at 37 °C. The CA16 virus strain was isolated from the clinical sample and expanded in RD cells. *Escherichia coli* (*E. coli*) TOP10F’ strain was used for the transformation of recombinant phagemid (pComb3XSS vector) containing scFv genes. The *Enterovirus* A71 strain used in the cross-reactivity assay was designated as *Enterovirus* 71/4643/TW/98.

### 4.3. Preparation of CA16-Infected Lysate

RD cells were infected with the CA16 virus at a multiplicity of infection (MOI) of 10 and were incubated for 6 h. After the infected-cell culture, the cells were collected, suspended, and lysed in a buffer containing 50 mM Tris-HCL (pH = 7.4), 150 mM sodium chloride (NaCl), 1% Triton X-100, and a protease inhibitor cocktail (Roche, Mannheim, Germany). After lysis, the supernatant containing the CA16 virus was obtained, and the cell debris was separated and pulled out by spinning. The collected supernatant as CA16-infected lysate was used for hen vaccination, enzyme-linked immunosorbent assay (ELISA) and Western blot.

### 4.4. Hen Vaccination

The white leghorn laying-hen (*Gallus domesticus*) was vaccinated with 100 µg CA16-infected lysate in an equal amount of Freund’s complete adjuvant (Sigma-Aldrich, Inc., St. Louis, MO, USA) by intramuscular injection. After one week, seven additional vaccinations with incomplete adjuvant were carried out following periods of 7 days each and then, two weeks after the last vaccination, the hen was sacrificed. The vaccinating hen was caged in a well-ventilated room at the Taipei laboratory animal center, and followed up daily. Hen eggs were collected every week after each vaccination and kept at 4 °C until partial purification and characterization of the polyclonal immunoglobulin yolk antibodies. The two adjuvants and virus-infected lysate were kept at 4 °C and −20 °C, respectively, until the vaccination was finished.

### 4.5. Hen IgY Purification from Egg Yolk

The yolk fraction containing specific immunoglobulin yolk against proteins in CA16-infected lysate was prepared from vaccinated eggs using the tris-buffered saline (TBS, pH = 7.4) and dextran sulfate method. In brief, the yolk’s membrane was pierced and the whole yolk was drawn by the syringe and dispersed into a 50-mL Falcon tube. The 10 mL of the transferred yolk was diluted with 40 mL of TBS and mixed thoroughly by vortexing. The mixture was allowed to stand at room temperature (RT) for 30 min. After incubation, the mixture was spun for 20 min at 10,000× *g* at RT. The supernatant was collected into a new 50-mL tube. Dextran sulfate (*w*/*v*) was added per milliliter (mL) of supernatant. After subsequent mixtures with calcium chloride (*w*/*v*) per mL, 20 g of anhydrous sodium sulfate, and 36% sodium sulfate solution, respectively, the final product was left to stand so that white precipitate could form. After centrifugation, the pellet was redissolved in 5 mL of the TBS. The suspended IgY was preserved with 0.05% sodium azide and kept at −20 °C for further characterization. 

### 4.6. Titration of IgY Antibodies

The antibody titer was tested by an ELISA protocol as described in the article [32] with little changes. The specific binding ability of IgY against proteins in CA16-infected lysate was demonstrated as follows. A microwell ELISA plate was coated with 25 µL of CA16-infected lysate and BSA per well. The specific IgY antibodies (pre-vaccination and eighth vaccination) diluted from 1:500 to 1:1,024,000 were reacted with the coated immunogens. The same volume (25 µL) of donkey horseradish peroxidase-conjugated anti-chicken IgY antibodies (diluted 1:10,000 in 5% skimmed milk) and freshly diluted (buffer A and B) substrate solution, 3,3′,5,5′-tetramethylbenzidine (TMB), were used for secondary antibodies and substrate, respectively. The optical density of the mixture was determined by a microplate ELISA reader at 450 nm wavelength.

### 4.7. RNA Extraction and cDNA Synthesis

After final immunization with CA16-infected lysate, the hen was sacrificed for total RNA extraction from the isolated spleen. The TriZol reagent (Invitrogen, Carlsbad, CA, USA) was used. Briefly, 200 µL chloroform reagent was added to 1 mL TriZol solution and mixed gently. After incubation for 3 min at RT, it was centrifuged for 15 min at 12,000× *g* at 4 °C. Then the aqueous part was collected and mixed with isopropanol for 10 min. After centrifugation, the supernatant was removed and the pellet was washed with cold 75% ethanol. After another centrifugation, the RNA pellet was air-dried for 10 min and then re-suspended in RNase-free water. To convert cDNA from mRNA, the isolated total RNA was treated with oligo (dT)_18_ primer. The sample mixture was processed according to the protocol described by the authors [21].

### 4.8. Preparation of Hen scFv Gene

To prepare the recombinant gene, we amplified the variable domains of the light chain (V_L_) and heavy chain (V_H_), using CSCVHo-F (5′-GGTCAGTCCTCTAGATCTTCCGCCGTGACGTTGGACGAG-3′) and CSCG-B (5′-CTGGCCGGCCTGGCCACTAGTGGAGGAGACGATGACTTCGGTCC-3′) primers for V_H_ with short-linkers (V_H-_S), CSCVHo-FL (5′-GGTCAGTCCTCTAGATCTTCCGGCGGTGGTGGCAGCTCCGGTGGTGGCGGTTCCGCCGTGACGTTGGACGAG-3′) and CSCG-B primers for V_H_ with long-linkers (V_H-_L), and the CSCVK (5′-GTGGCCCAGGCGGCCCTGACTCAGCCGTCCTCGGTGTC-3′) and CKJo-B (5′-GGAAGATCTAGAGGACTGACCTAGGACGGTCAGG-3′) primers for V_L_, in the first round of the polymerase chain reaction (PCR). Briefly, in each PCR reaction, 60 pmole forward primer, 60 pmole reverse primer, 1 μL converted cDNA, and 50 μL 2× Taq DNA polymerase master mix red were added into a 0.2 mL tube; the final volume was adjusted to 100 μL with double distilled water (ddH_2_O). The mixture was subjected to the following PCR conditions: 94 °C for 5 min, then 30 cycles of 94 °C for 15 s, 56 °C for 15 s, and 72 °C for 90 s. The last step included incubation at 72 °C for 10 min. The PCR products were examined on 1% agarose gel. The products of each reaction were pooled and concentrated by ethanol precipitation. Concentrated products were separated on 2% agarose gel electrophoresis. To amplify the full scFv gene, the second round of PCR was performed using the CSC-F (5′-GAGGAGGAGGAGGAGGAGGTGGCCCAGGCGGCCCTGACTCAG-3′) and CSC-B (5′-GAGGAGGAGGAGGAGGAGGAGCTGGCCGGCCTGGCCACTAGTGGAGG-3′) primers mixed with the appropriate first-round purified PCR products. In each 0.2 mL PCR tube reaction, 60 pmole primer (forward), 60 pmole primer (reverse), 100 ng V_H-S_ or V_H-L_ products, 100 ng V_L_ products and 50 μL 2× Taq DNA polymerase master mix red were mixed and the final volume was adjusted to 100 μL ddH_2_O. The mixture was run under the following conditions: 94 °C for 5 min, then 25 cycles of 94 °C for 15 s, 56 °C for 15 s and 72 °C for 2 min. Finally, it was incubated at 72 °C for 10 min. After amplification, the PCR products were analyzed on 2% agarose gel electrophoresis to check the purity. Eventually, the correct band-size was cut out, approximately 800 bps for scFv-S or approximately 850 base pairs (bps) for scFv-L, and purified using gel extraction.

### 4.9. Cloning of Hen scFv Gene into pComb3X DNA Vector

Before antibody library construction, the purified scFv PCR products and pComb3X plasmid DNA vector were digested using the *Sfil* restriction enzyme for cloning. The digestion steps were carried out as described in a previous study [38]. The mixture was concentrated by the ethanol-precipitation method as described above. The scFv products were gel-purified, as was the pComb3X DNA vector using an Advanced Gel Extraction System (Viogene BioTek Corp, New Taipei City, Sijhih, Taiwan). The purity of the products was checked by running agarose gel electrophoresis. In the ligation reaction, the following was added into a new Eppendorf tube: *Sfil*-digested and purified pComb3X DNA, *Sfil*-digested and purified scFv-S or scFv-L, 5× ligase buffer, and DNA ligase, which were mixed, and ddH_2_O was added to a final volume. The mixtures were incubated overnight at 23 °C. 

### 4.10. Construction of Phage-Displaying scFv Libraries

To generate scFv antibody libraries, the hen-derived scFv antibody libraries were prepared for subsequent use according to the published article with minimal changes [39]. In brief, the isolated total RNA from the hen spleen was used as a starting material to synthesize into cDNA using reverse transcriptase. The cDNA molecule was used to amplify the variable regions of the light chain (V_L_) and heavy chain (V_H_). The two products (V_L_ and V_H_) were connected by a 7- or 18-amino acids flexible peptide linker to make a full-length scFv with a short-linker or a long-linker through an overlapping extension polymerase chain reaction. After digestion with *SfiI* (New England BioLabs, Ipswich, MA, USA) at 50 °C for 5 min, the genes of scFv were cloned into a phagemid vector (pComb3X), and the recombinant DNA products were transformed into electrocompetent *Escherichia coli* (ER2738 strain) by electroporation (Bio-Rad Micropulser, Hercules, CA, USA). Then, the mixture was transferred into a pre-chilled cuvette and electroporated at 3.0 kV (Bio-Rad Micropulser, Hercules, CA, USA). The transformation step was repeated three times for all ligation products. After streaking a small amount on an LB agar plate, the total number of transformants was calculated by counting the number of colonies, multiplying the culture volume, and dividing the plating volume to determine the library size. The remaining culture was mixed with 100 mL of super broth (SB). After 2 h culture, the VCS-M13 helper phage (10^12^ PFU) was added to help package the phages. The following day, the culture was centrifuged at approximately 4000× *g* for 10 min to collect the supernatant containing recombinant phages. The phages were obtained by precipitation using 4% (*w*/*v*) polyethylene glycol 8000 (PEG-8000) and 3% (*w*/*v*) sodium chloride (NaCl) and incubated on ice for at least 30 min after thorough dissolving. After two more steps and further centrifugation, the precipitated phages were resuspended in PBS reconstituted by 1% bovine serum albumin (BSA) and plus 20% glycerol as preservatives and stored at −20 °C.

### 4.11. Bio-Panning of scFv-Binding Protein Antibodies and Selection

To screen out specific scFv-binding protein antibodies, the CA16-infected lysate (100 µg/mL) was coated on ELISA well plates (Becton Dickinson, San Jose, CA, USA) and incubated at 4 °C overnight. The plate was blocked for 2 h at 37 °C with 3% bovine serum albumin (BSA) dissolved in phosphate-buffered saline (PBS), then washed three times with PBST (plus 0.05% Tween-20). Next, 10^12^ PFU (plaque-forming unit) phage was added and incubated for 2 h at 37 °C. After phage binding, the ELISA well-plate was washed 10 times at an interval of 30 vigorous pipettings up and down with filtered PBST. The bound phages were eluted with 0.2 M glycine-HCl pH = 2.2 elution buffer (reconstituted with 1 mg/mL BSA) in each well, and the eluate was immediately neutralized with 2 M Tris-base buffer. The eluted phages were amplified as narrated in the published paper [40]. Also, the scFv antibodies were selected from a constructed phage library. After the fifth round of selecting the specific binders, the total phagmid DNA was extracted from the bacterial pellet and transformed into competent TOP10F′ *Escherichia coli* for scFv expression and purification. The transformed cells were cultured on a Luria Bertani (LB) agar plate with 50 μg/mL of ampicillin (Amp) and incubated overnight at 37 °C. The randomly selected colonies from the LB agar plate were grown in super broth consisting of 1 M MgCl_2_ and 50 μg/mL of Amp, and incubated at 37 °C for 8 h, at which time 1 mM isopropyl β-D-1-thiogalactopyranoside (IPTG, Thermo Fischer Scientific, Waltham, MA, USA) was added for overnight induction of protein expression [41]. After incubation, the bacteria were collected and processed as previously described [42]. Briefly, the induced cells were pelleted by spinning at approximately 3000× *g* for 10 min at 4 °C and the bacterial pellet was re-suspended in a pH = 7.4 histidine (His)-binding buffer with 6 molar (M) urea (20 mM sodium phosphate, 0.5 M NaCl, 20 mM imidazole), and sonicated on ice at a condition of 85% amplitude, 20 s vibration, and then 20 s rest for 6 cycles. The cell debris was removed by spinning at approximately 15,000× *g* for 10 min at 4 °C. The supernatant was mixed with nickel beads (Ni^2+^-sepharose, GE Healthcare Biosciences AB, Uppsala, Sweden) to pull down the His-fused single-chain fragment variable proteins according to the manufacturer’s protocol. The purified scFv proteins which contained the His-tag were checked for purity by Coomassie staining of SDS-gels and Western blot.

### 4.12. SDS-PAGE and Immunoblotting Techniques

The protein gel electrophoresis described by Backman and Persson [43] was carried out using a 4% stacking gel and 15% resolving gel. After virus-infected lysate preparation, the viral proteins were separated by sodium dodecyl sulfate–polyacrylamide gel electrophoresis (SDS-PAGE). The virus-infected lysate (30 µg) and BSA (30 µg) were pre-incubated with SDS sample buffer at a 1:1 dilution without β-mercaptoethanol (β-ME); the sample mixture was boiled in a Bio-Rad PCR machine at 95 °C for 10 min. The final volumes of 5.7 µL, 3.9 µL, and 5 µL for CA16- and EV71-infected lysate and BSA, respectively, were loaded into each well of the gels, and run at 80 volts for at least 2 h. The proteins in CA16-infected lysate were either stained with Coomassie blue or electroblotted onto a polyvinylidene difluoride (PVDF) membrane according to the manufacturer’s manual. The transferred proteins on the membrane were blocked with 5% skimmed milk in PBST (1× phosphate-buffered saline plus 0.05% tween-20) for at least 1 h. This was then incubated at room temperature with specific scFv antibodies at specified dilutions (30 µg/mL) in PBST for another 1 h. Bound antibodies were detected using mouse anti-human influenza hemagglutinin (HA) tag antibodies, the secondary antibody for 1 h, and then followed by horseradish peroxidase-conjugated rabbit anti-mouse IgG (Jackson ImmunoResearch Laboratories, Inc., West Grove, PA, USA), antibody for an additional 1 h. The blotted membrane was developed for 15 min with 3,3′-diaminobenzedine (DAB) substrate (Bio-Rad Laboratories Inc., Vlsalla, CA, USA) according to the manufacturer’s instructions.

### 4.13. Enzyme-Linked Immunosorbent Assay (ELISA)

Virus-infected lysate and BSA (100 μg/mL) were coated in a 96-half-area-well plate (Becton Dickinson, San Jose, CA, USA). The 96-well plate was incubated for 1 h at 37 °C and blocked with 5% skimmed milk (150 µL/well) for another 1 h at 37 °C. After blocking, the wells were washed six times with 150 µL PBST [44]. The plates were incubated for 1 h at 37 °C with primary antibodies (single-chain and immunoglobulin yolk antibodies) at 30 µg/mL and 3000× dilutions, respectively. After incubation and washing, a 5000× dilution of the secondary antibody was added into each well and incubated for 1 h at 37 °C. Each well was then washed six times with PBST. The plate probed with scFv antibody was further incubated with rabbit anti-mouse IgG HRP (tertiary antibody) for 1 h at 37 °C. For the IgY antibody titer, the serial dilution was carried out at 500× as the starting dilution and incubated for 1 h at 37 °C after the well plate was blocked with 5% skimmed milk. Twenty-five µL of TMB was added to each well until the color became strong blue. Then, 1 N HCl was added to stop the reaction, and the color changed from blue to yellow, signifying no further reaction. The optical density was measured at 450 nm by an ELISA plate reader. All ELISA results were represented as the means plus or minus the standard deviation (SD) of duplicated determinations.

### 4.14. In Vitro scFv-Binding Protein Neutralization Assay

The neutralization assay measured the antibody’s ability to inhibit the cytopathic effects (CPEs) that were induced by viruses. In brief, 96-well tissue culture plates were seeded with 3 × 10^4^ cells per well in DMEM medium with 10% fetal bovine serum (FBS). After 24 h of incubation at 37 °C, human RD cells were infected with the virus at a multiplicity of infection of 10 per cell. After adsorption, the infected cells were covered with medium containing 2% FBS and antibody at different concentrations. The infected cells were further incubated at 37 °C for 24 h. The plates were fixed with 0.5% formaldehyde and then stained with 0.1% crystal violet. The density of the well was measured at 570 nanometers (nm). Each experiment was performed in triplicate and repeated at least twice. The 50% inhibitory concentration (IC_50_) was calculated. The formula IC_50_ = [(Y − B)/(A − B)] × (H − L) + L] was used. Y represents half of the mean optical density at 570 nm (OD_570_) for the control cells without the antibody. B represents the mean OD_570_ of wells with the antibody dilution nearest to and below Y. A represents the mean OD_570_ of wells with the antibody dilution nearest to and above Y. L and H are the antibody concentrations at B and A, respectively.

### 4.15. Statistical Analysis

The statistical data were analyzed using GraphPad Prism software (version 8.0). The results were presented as the means ± standard deviation (SD) for the absorbance reading of the enzyme-linked assay. All ELISA experiments were run in duplicate and triplicate.

## 5. Conclusions

In summary, our study shows that IgY-binding protein antibodies were elicited in a laying hen in response to vaccination with CA16-infected lysate. This implies that viral proteins can be the potential target for developing vaccines, as shown in cross-reactivity assays. The scFv-binding protein antibody was screened from laying hen-derived single-chain antibody libraries. This scFv-binding protein S1 has shown that it can also potentially be used as a therapeutic agent, as observed in the present study. The scFv antibody can accurately bind to proteins of genetically closely related enteroviruses because the scFv antibody shows the cross-reactive property. Moreover, IgY and scFv antibodies displayed different abilities to recognize the proteins in virus-infected lysate in Western blot analysis. The IgY antibodies not only detected one protein with a molecular weight close to 75 kDa, which could be 3CD (polyprotein) in CA16-infected lysate, but also recognized a protein close to 37 kDa, which was speculated to be VP1. On the other hand, scFv recognized similar protein bands patterns as did the IgY antibody in EV71-infected lysate with molecular weights close to 37 and 75 kDa. Taken together, scFv-binding protein S1 is not specifically against proteins in CA16-infected lysate but is a cross-reactive antibody against EV71, while the IgY antibody has the properties of being both a specific and a neutralizing antibody to CA16, which creates potential for investigation tools and vaccine development for CA16 and EV71 viruses.

## Figures and Tables

**Table 1:** White laying-hen vaccination schedule.

	Amount of Antigen (μg, CA16-Infected Lysate)	Volume of 1× PBS (μL)	Adjuvants (500 μL)
1st-vaccination	100	500	Complete freund’s adjuvant
2nd-vaccination	100	500	Incomplete freund’s adjuvant
3rd-vaccination	100	500	Incomplete freund’s adjuvant
4th-vaccination	100	500	Incomplete freund’s adjuvant
5th-vaccination	100	500	Incomplete freund’s adjuvant
6th-vaccination	100	500	Incomplete freund’s adjuvant
7th-vaccination	100	500	Incomplete freund’s adjuvant
8th-vaccination	100	500	Incomplete freund’s adjuvant

**Table 2:** Amino acid mutation rates of the light- and heavy-chain genes of the scFv antibody.

ScFv Clone	Region	Diff/totl (%) *								
		FR1	FR2	FR3	FR4	Total FRs	CDR1	CDR2	CDR3	Total CDRs
**ScFv-binding protein S1**	**V_L_**	1/20 (5)	1/16 (6.25)	6/32 (18.75)	0/10 (0)	8/78 (10.25)	7/13 (53.85) ^a^	2/7 (28.57)	4/11 (36.36)	13/31 (41.94) ^a^
	**V_H_**	3/30 (10)	0/14 (0)	3/32 (9.38)	0/11 (0)	6/87 (6.90)	2/5(40)	8/17 (47.06)	12/15 (80) ^a^	22/37 (59.46) ^a^

* Diff/totl (%): number of differences/total number (percentage); ^a^: insertion mutation rate; CDRs: complementarity determining regions; FRs: framework regions; V_L_: variable domains of the light chain; V_H_: variable domain of the heavy chain.

## Data Availability

Not applicable.

## Abbreviations

SCFV	Single-chain variable fragment
TMB	3,3′, 5,5′-tetramethylbenzidene
PFU	Plaque-forming unit
SD	Standard deviation
OD	Optical density
µg/mL	Microgram per milliliter
%	Percentage
M	Molar
MgCl_2_	Magnesium chloride
mg/mL	Milligram per milliliter
*w*/*v*	Weight per volume
pmole	Picomole
µL	Microliter
ng	Nanogram
°C	Degree Celsius
PVDF	Polyvinylidene fluoride

## References

[1] Yu W., Xu H., Yin C. (2016). Molecular epidemiology of human coxsackievirus A16 strains. Biomed. Rep..

[2] Mao Q., Wang Y., Yao X., Bian L., Wu X., Xu M., Liang Z. (2014). Coxsackievirus A16: Epidemiology, diagnosis, and vaccine. Hum. Vaccin. Immunother..

[3] Xu W., Liu C.-F., Yan L., Li J.-J., Wang L.-J., Qi Y., Cheng R.-B., Xiong X.-Y. (2012). Distribution of enteroviruses in hospitalized children with hand, foot and mouth disease and relationship between pathogens and nervous system complications. Virol. J..

[4] Wang C.-Y., Lu F.L., Wu M.-H., Lee C.-Y., Huang L.-M. (2004). Fatal coxsackievirus A16 infection. Pediatr. Infect. Dis. J..

[5] Bendig J., Fleming D. (1996). Epidemiological, virological, and clinical features of an epidemic of hand, foot, and mouth disease in England and Wales. Commun. Dis. Rep. CDR Rev..

[6] Chia M.-Y., Chiang P.-S., Chung W.-Y., Luo S.-T., Lee M.-S. (2014). Epidemiology of enterovirus 71 infections in Taiwan. Pediatr. Neonatol..

[7] Zhao K., Han X., Wang G., Hu W., Zhang W., Yu X.-F. (2011). Circulating coxsackievirus A16 identified as recombinant type A human enterovirus, China. Emerg. Infect. Dis..

[8] Zhang Y., Zhu Z., Yang W., Ren J., Tan X., Wang Y., Mao N., Xu S., Zhu S., Cui A. (2010). An emerging recombinant human enterovirus 71 responsible for the 2008 outbreak of hand foot and mouth disease in Fuyang city of China. Virol. J..

[9] Mao Q.-y., Wang Y., Bian L., Xu M., Liang Z. (2016). EV71 vaccine, a new tool to control outbreaks of hand, foot and mouth disease (HFMD). Expert Rev. Vaccines.

[10] Luo S., Wu F., Ye X., Fu T., Tao J., Luo W., Wang Y., Jia J., Lou L. (2019). Safety comparison of two enterovirus 71 (EV71) inactivated vaccines in Yiwu, China. J. Trop. Pediatr..

[11] Tan Y., Chu J. (2017). Sinovac EV71 vaccine: The silver bullet for hand, foot and mouth disease—Or not. J. Public Health Emerg..

[12] Marston H.D., Paules C.I., Fauci A.S. (2018). Monoclonal antibodies for emerging infectious diseases—Borrowing from history. N. Engl. J. Med..

[13] Rudolph H., Schroten H., Tenenbaum T. (2016). Enterovirus Infections of the Central Nervous System in Children: An Update. Pediatr. Infect. Dis. J..

[14] Kuroda M., Niwa S., Sekizuka T., Tsukagoshi H., Yokoyama M., Ryo A., Sato H., Kiyota N., Noda M., Kozawa K. (2015). Molecular evolution of the VP1, VP2, and VP3 genes in human rhinovirus species C. Sci. Rep..

[15] Lewis J.K., Bothner B., Smith T.J., Siuzdak G. (1998). Antiviral agent blocks breathing of the common cold virus. Proc. Nat. Acad. Sci. USA.

[16] Strauss M., Filman D.J., Belnap D.M., Cheng N., Noel R.T., Hogle J.M. (2015). Nectin-like interactions between poliovirus and its receptor trigger conformational changes associated with cell entry. J. Virol..

[17] Bird R.E., Hardman K.D., Jacobson J.W., Johnson S., Kaufman B.M., Lee S.-M., Lee T., Pope S.H., Riordan G.S., Whitlow M. (1988). Single-chain antigen-binding proteins. Science.

[18] Huston J.S., Levinson D., Mudgett-Hunter M., Tai M.-S., Novotný J., Margolies M.N., Ridge R.J., Bruccoleri R.E., Haber E., Crea R. (1988). Protein engineering of antibody binding sites: Recovery of specific activity in an anti-digoxin single-chain Fv analogue produced in Escherichia coli. Proc. Nat. Acad. Sci. USA.

[19] Xu X., Zhang R., Chen X. (2017). Application of a single-chain fragment variable (scFv) antibody for the confirmatory diagnosis of hydatid disease in non-endemic areas. Electron. J. Biotechnol..

[20] Krag D.N., Shukla G.S., Shen G.-P., Pero S., Ashikaga T., Fuller S., Weaver D.L., Burdette-Radoux S., Thomas C. (2006). Selection of tumor-binding ligands in cancer patients with phage display libraries. Cancer Res..

[21] Unkauf T., Miethe S., Fühner V., Schirrmann T., Frenzel A., Hust M. (2016). Generation of recombinant antibodies against toxins and viruses by phage display for diagnostics and therapy. Protein Targeting Compounds.

[22] Corti D., Voss J., Gamblin S.J., Codoni G., Macagno A., Jarrossay D., Vachieri S.G., Pinna D., Minola A., Vanzetta F. (2011). A neutralizing antibody selected from plasma cells that binds to group 1 and group 2 influenza A hemagglutinins. Science.

[23] Yokota T., Milenic D.E., Whitlow M., Schlom J. (1992). Rapid tumor penetration of a single-chain Fv and comparison with other immunoglobulin forms. Cancer Res..

[24] Pyo H.-M., Kim I.-J., Kim S.-H., Kim H.-S., Cho S.-D., Cho I.-S., Hyun B.-H. (2009). Escherichia coli expressing single-chain Fv on the cell surface as a potential prophylactic of porcine epidemic diarrhea virus. Vaccine.

[25] Harmsen M., Fijten H., Engel B., Dekker A., Eblé P. (2009). Passive immunization with llama single-domain antibody fragments reduces foot-and-mouth disease transmission between pigs. Vaccine.

[26] Bird C.R., Thorpe R. (2009). Purification of immunoglobulin Y (IgY) from chicken eggs. The Protein Protocols Handbook.

[27] Deng X.K., Nesbit L.A., Morrow K.J. (2003). Recombinant Single-Chain Variable Fragment Antibodies Directed against Clostridium difficile Toxin B Produced by Use of an Optimized Phage Display System. Clin. Diagn. Lab. Immunol..

[28] Fehrsen J., Wemmer S., van Wyngaardt W., Hust M., Lim T.S. (2018). Construction of Chicken Antibody Libraries. Phage Display: Methods and Protocols.

[29] Yang Q., Ding J., Cao J., Huang Q., Hong C., Yang B. (2015). Epidemiological and etiological characteristics of hand, foot, and mouth disease in Wuhan, China from 2012 to 2013: Outbreaks of coxsackieviruses A10. J. Med. Virol..

[30] Nilvebrant J., Sidhu S.S. (2018). Construction of synthetic antibody phage-display libraries. Phage Display.

[31] Da Silva M.C., Schaefer R., Gava D., Souza C.K., da Silva Vaz I., Bastos A.P., Venancio E.J. (2018). Production and application of anti-nucleoprotein IgY antibodies for influenza A virus detection in swine. J. Immunol. Methods.

[32] Lee C.-H., Leu S.-J., Lee Y.-C., Liu C.-I., Lin L.-T., Mwale P.F., Chiang J.-R., Tsai B.-Y., Chen C.-C., Hung C.-S. (2018). Characterization of chicken-derived single chain antibody fragments against venom of Naja naja atra. Toxins.

[33] Cai Y., Liu Q., Huang X., Li D., Ku Z., Zhang Y., Huang Z. (2013). Active immunization with a Coxsackievirus A16 experimental inactivated vaccine induces neutralizing antibodies and protects mice against lethal infection. Vaccine.

[34] Liu F., Liu Q., Cai Y., Leng Q., Huang Z. (2011). Construction and characterization of an infectious clone of coxsackievirus A16. Virol. J..

[35] Li T.-W., Cheng S.-F., Tseng Y.-T., Yang Y.-C., Liu W.-C., Wang S.-C., Chou M.-J., Lin Y.-J., Wang Y., Hsiao P.-W. (2016). Development of single-chain variable fragments (scFv) against influenza virus targeting hemagglutinin subunit 2 (HA2). Arch. Virol..

[36] Du R., Mao Q., Hu Y., Lang S., Sun S., Li K., Gao F., Bian L., Yang C., Cui B. (2019). A potential therapeutic neutralization monoclonal antibody specifically against multi-coxsackievirus A16 strains challenge. Hum. Vaccines Immunother..

[37] Zhang W., Dai W., Zhang C., Zhou Y., Xiong P., Wang S., Ye X., Liu Q., Zhou D., Huang Z. (2018). A virus-like particle-based tetravalent vaccine for hand, foot, and mouth disease elicits broad and balanced protective immunity. Emerg. Microbes Infect..

[38] Zhang H., Wang X., Li X., Ma Z., Feng R. (2018). Construction, expression, and characterization of a single-chain variable fragment (ScFv) antibody targeting to the encephalomyocarditis virus. J. Med. Virol..

[39] Mwale P.F., Lee C.-H., Leu S.-J., Lee Y.-C., Wu H.-H., Lin L.-T., Lin T.E., Huang Y.-J., Yang Y.-Y. (2019). Antigenic epitopes on the outer membrane protein A of Escherichia coli identified with single-chain variable fragment (scFv) antibodies. Appl. Microbiol. Biotechnol..

[40] Leu S.-J., Lee Y.-C., Shih N.-Y., Huang I.-J., Liu K.-J., Lu H.-F., Huang S.-Y., Yang Y.-Y. (2010). Generation and characterization of anti-α-enolase single-chain antibodies in chicken. Vet. Immunol. Immunopathol..

[41] Terpe K. (2006). Overview of bacterial expression systems for heterologous protein production: From molecular and biochemical fundamentals to commercial systems. Appl. Microbiol. Biotechnol..

[42] Tikunova N., Morozova V. (2009). Phage display on the base of filamentous bacteriophages: Application for recombinant antibodies selection. Acta Nat..

[43] Backman L., Persson K. (2018). The No-Nonsens SDS-PAGE. Methods Mol. Biol..

[44] Engvall E., Perlmann P. (1971). Enzyme-linked immunosorbent assay (ELISA). Quantitative assay of immunoglobulin G. Immunochemistry.

